# Effect of Amniotic Membrane Dressing on Pain and Healing of Palatal Donor Site: A Randomized Controlled Trial

**Published:** 2020

**Authors:** Z. Kadkhoda, A. Tavakoli, S. Chokami Rafiei, F. Zolfaghari, S. Akbari

**Affiliations:** 1 *Associate Professor, Department of Periodontics, School of Dentistry, Tehran University of Medical Sciences, Tehran, Iran*; 2 *Assistant Professor, Iranian Tissue Bank Research Center, Tehran University of Medical Sciences, Tehran, Iran *; 3 *Assistant Professor, Department of Periodontics, School of Dentistry, Qazvin University of Medical Sciences, Qazvin, Iran*; 4 *Postgraduate Student, Department of Periodontics, School of Dentistry, Tehran Medical Sciences, Islamic Azad University, Tehran, Iran*; 5 *Assistant Professor, Department of Periodontics, School of Dentistry, Tehran University of Medical Sciences, Tehran, Iran*

**Keywords:** Amniotic membrane, Biological dressing, Free gingival graft, Post-operative pain, Wound healing, Regeneration

## Abstract

**Background:**

Free gingival graft is the most commonly practiced predictable technique for gingival augmentation.

**Objective:**

To assess the effectiveness of human amniotic membrane, a biological dressing, on wound healing and post-operative pain after its application on the palatal donor site after free gingival graft surgery.

**Methods:**

Of 27 eligible patients, 15 were randomized into a test group and received human amniotic membrane dressing sutured over their palatal donor site; 12 were randomized into a control group in whom the palatal donor site was only sutured. Standard clinical photographs were taken at 7, 14, and 21 days post-operatively and evaluated by 3 periodontists. The pain score at the donor site was assessed by a visual analog score; the number of analgesics taken was also recorded.

**Results:**

The mean color match scores were higher in the test group than the control group at 14 (p<0.01) and 21 days after surgery (p=0.02). The difference in tissue texture (p=0.01) and inflammation (p=0.02) between the two groups was only significant on day 14 (p<0.05). The pattern of pain relief was better in the test group compared with the control group, especially in first days, although the differences were not significant in terms of the number of analgesics taken or the pain score.

**Conclusion:**

Application of human amniotic membrane can accelerate wound healing and may decrease post-operative pain and discomfort by a limited amount.

## INTRODUCTION

Free gingival graft is the most commonly practiced predictable technique for gingival augmentation. It is used to increase the keratinized gingival width and depth of the vestibule, prevent gingival recession, improve plaque control and esthetics, and decrease or eliminate root hypersensitivity [1, 2]. This surgical procedure, however, results in an open wound in the palate that heals within 2–4 weeks [3]. The open wound causes pain and discomfort for patients after surgery. In cases requiring multiple gingival grafts, a minimum of 4-week interval is required between the surgical procedures to allow complete healing of the palate [4].

Various materials and methods have been used to accelerate palatal wound healing including laser therapy [5] and topical application of ozonated oil [6], platelet-rich fibrin [7], and medicinal plant extracts [8].

Evidence shows lower infection rate and degree of shrinkage induced by scarring in wounds covered with biological dressings compared with those that remain exposed or are covered with synthetic materials [9-11]. Therefore, biological dressings that do not undergo degradation for a certain period might be beneficial for wound healing.

The amniotic membrane (AM) is a biological dressing with many therapeutic effects [12-16]. There is a growing interest to use this material for periodontal tissue repair [17]. AM is the most internal layer of the placenta, which consists of three layers: an epithelial monolayer, a thick basement membrane, and a collagen-rich underlying stroma. It has no nerves, muscles or lymphatic vessels, and can be easily separated from the underlying chorion.

Studies have shown that AM induces re-epithelialization and angiogenesis and decreases the inflammatory response. It also has antibacterial properties and contains growth factors such as tumor growth factor-alpha and fibroblast growth factor-beta as well as mesenchymal stem cells with different differentiation potential [18, 19].

AM is also a suitable material for allografts because of its low immunogenicity. It has anti-inflammatory properties, protects the wound and decreases scar tissue formation. AM has been used for decades in various clinical settings, including ophthalmology and wound care [20]. It decreases the level of pain and inflammation and risk of infection since its stromal surface adheres to the wound surface, covers the exposed free nerve endings in the wound area, protects the wound surface from trauma and external stimuli, and minimizes the protein and fluid loss from the wound [21].

This study was conducted to assess the effect of lyophilized AM, as a biological dressing, applied on palatal donor site in free gingival graft surgery. The objective was to find a proper dressing for wound coverage to decrease pain and discomfort following the surgery.

## MATERIALS AND METHODS

STUDY DESIGN

This study was a randomized controlled clinical trial. Allocation of patients to the test and control groups was carried out using sealed opaque coded envelopes that were opened right before the surgery. 

Study Population

Thirty-four patients requiring gingival augmentation referred to the Department of Periodontology, School of Dentistry, Tehran University of Medical Sciences, were examined to assess their eligibility for participation in this study. Of 34 patients, 27 (14 males and 13 females, aged 18–70 years, mean age of 54 years) met the inclusion criteria.

The exclusion criteria were: O’Leary’s plaque index >20% [22], smoking, uncontrolled diabetes mellitus, immunocompromised patients or those taking immunosuppressive drugs, pregnant women, and patients suffering from mucosal and skin diseases. The surgical procedures were thoroughly explained to all patients who signed informed written consent forms. The research protocol was registered with the Iranian Registry of Clinical Trials, IRCT2016021321069N1. The study protocol was approved by the Ethics Committee of School of Dentistry, Tehran University of Medical Sciences. 

Preparation of the Human AM Graft

The AM was procured from the placenta of healthy pregnant women undergoing elective Cesarean section. The pregnant women were excluded if they had immunodeficiency, transmissible diseases or infectious diseases. The AM graft was prepared and preserved as described by Kim and Tseng [23]. The human placentas were obtained shortly after the deliveries. Under a hood with laminar air flow, the placenta was washed with normal saline to eliminate blood clots. The AM was readily separated from the chorion by blunt dissection. The membrane was then washed with phosphate buffered saline containing 1000 U/mL penicillin, 20 mg/mL streptomycin, and 2.5 µg/mL amphotericin B three times. The membrane was freeze-dried at -80 °C, packed in a two-layer polyethylene bag and transferred to the Iranian Atomic Energy Agency in a radiation box for sterilization. 

Surgical Technique

**Figure 1:**
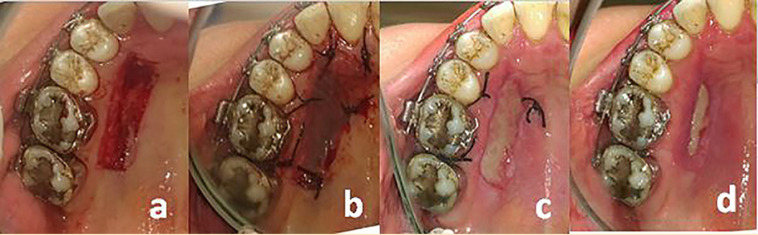
(a) Appearance of palatal site after the graft harvested, (b) application of AM, clinical appearance after surgery on (c) day 7, and (d) day 14

**Figure 2:**
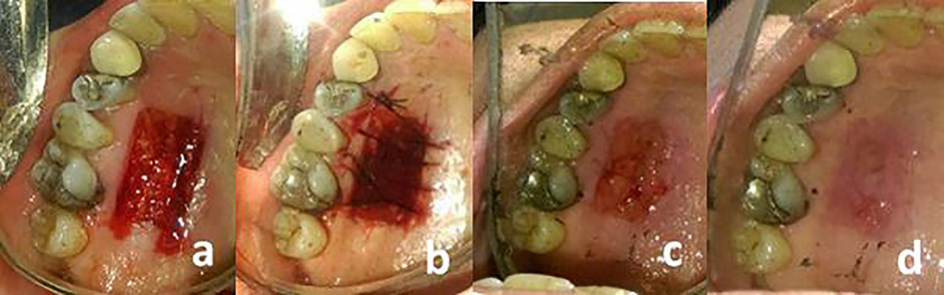
(a) Palatal site after harvesting the graft, (b) suturing, (c) clinical appearance after surgery on day 7, and (d) day 14

Initial periodontal therapy was performed for all patients. An experienced periodontist performed all the surgical procedures. The procedures were carried out under local anesthesia according to the most commonly used technique described by Sullivan and Atkins [24] and developed by Miller. After harvesting the graft, it was correctly positioned at the site, firmly adapted to the bed, stabilized with simple periosteal sutures and protected by periodontal dressing in both groups. The donor site was manually compressed with wet gauze for 1 min to achieve hemostasis in both groups. The AM was cut to a size slightly larger than the actual size of the wound by sterile scissors and was then immersed in normal saline to remain hydrated. In the test group, the AM was placed over the donor site and sutured such that the polyethylene surface was in contact with the wound (Fig 1). The control group donor site was sutured with 4-0 braided silk and protected with periodontal dressing (Fig 2). Patients were provided with post-operative instructions aiming to prevent mechanical trauma to the surgical site, which included discontinuation of tooth brushing and flossing around the surgical sites until the day of periodontal dressing removal (day 7). Amoxicillin (500 mg, TID, 7 days), nonsteroidal anti-inflammatory drugs (Gelofen, 400 mg), and 0.2% chlorhexidine digluconate mouthwash) BID, 14 days) were prescribed for all patients. All the patients were followed on days 7, 14, and 21 for further evaluation of the donor site.

Clinical Variables Examined

The patients were examined at days 7, 14, and 21 post-operatively. Standard photographs were taken of the donor site during the follow-up visits with a digital camera (Canon, Tokyo, Japan) under standard conditions from a distance of 20 cm and perpendicular to the palatal donor site. The photographs were observed by three independent and masked periodontists who were asked to express their opinion about the clinical healing of the donor sites. The inter-rater reliability was high (correlation coefficient: 0.85–0.90). The results of the stricter examiner were used for statistical analysis. The pattern of clinical healing of the palatal donor site was compared between the test and control groups by evaluating the degree of color match, tissue texture and inflammation. The color match of the donor site with the adjacent and contralateral palatal mucosa was evaluated using a visual analog scale (VAS). The VAS scores ranged from ‘0’ to ‘10’ where ‘0’ indicated “no color match” while ‘10’ indicated “excellent color match” [25]. The tissue texture of the donor site (similarity of the tissue texture of the palatal donor site to the adjacent tissue) was evaluated using a 4-point scale: ‘0’ indicated no similarity; ‘1,’ less than 50% similarity; ‘2,’ >50% similarity; and ‘3,’ complete similarity. Inflammation at the donor site was also assessed using a 4-point scale: ‘0’ showed no inflammation at the outer borders of the palatal wound; ‘1,’ mild inflammation; ‘2,’ moderate inflammation; and ‘3,’ severe inflammation.

Pain Assessment

During the 21 post-operative days, all the patients were requested to fill out a questionnaire regarding the number of non-steroidal anti-inflammatory analgesics they took for pain relief and a VAS score for pain between 0 and 10 (‘0’ indicated no pain; ‘1,’ minimal pain; and ’10,’ severe pain) [26]. Since the VAS pain scores reached zero during the first post-operative week, only the VAS pain scores during the first week were reported. 

Statistical Analysis

One-sample Kolmogorov-Smirnov test revealed that the variables studied did not have normal distribution. Therefore, Mann-Whitney U test was used for comparison of variables. A p value <0.05 was considered statistically significant.

## Results

The clinical healing of the surgical site was uneventful in all studied patients. Table 1 shows the color match, tissue texture and inflammation scores in the two groups. The mean VAS score for color match was higher in the test group on days 14 and 21 compared with that in the control group (p<0.001 and p꞊0.02, respectively). Tissue texture score (p꞊0.01) and inflammation score (p꞊0.02) were significantly different between the two groups only on day 14. 

**Table 1:** Mean±SD color match, tissue texture, and inflammation scores in the AM and control groups on days 7, 14, and 21 post-surgery

Variable	Baseline	7^th^ day	14^th^ day	21^st^ day
Color match				
AM group	0.00±0.00	6.60±1.12	8.87±0.74	9.87±0.35
Control group	0.00±0.00	5.75±0.97	7.92±0.79	9.25±0.62
p value	1.00	0.06	0.00	0.02
Tissue texture				
AM group	0.00±0.00	1.80±0.56	2.67±0.49	3.00±0.00
Control group	0.00±0.00	1.42±0.52	2.08±0.29	2.67±0.49
p value	1.00	0.14	0.01	0.15
Inflammation				
AM group	3.00±0.00	1.07±0.59	0.40±0.51	0.00±0.00
Control group	3.00±0.00	1.58±0.67	1.08±0.67	0.33±0.49
p value	1.00	0.05	0.02	0.15

Analgesics intake and pain scores decreased during the first week after surgery (Table 2). Pattern of pain relief, based on VAS pain scores, was better in the test group compared with the control group, especially in during the first days, although no significant difference was observed between the two groups. 

**Table 2:** Mean±SD number of analgesics taken by patients and the reported VAS pain scores within 7 days of surgery in the two groups

Variable	Day						
	1	2	3	4	5	6	7
Number of analgesics taken							
AM group	2.80±0.41	2.40±0.91	1.93±1.03	1.67±1.29	1.27±1.11	1.27±1.11	0.13±0.52
Control group	2.67±0.99	2.50±1.45	2.25±1.42	2.17±1.34	1.25±1.66	1.17±1.85	0.00±0.00
p value	0.90	0.49	0.40	0.35	0.61	0.37	0.79
Visual analog scale pain scores							
AM group	6.73±2.94	5.07±2.46	4.00±2.30	3.80±2.24	3.07±2.25	2.80±2.54	2.00±2.39
Control group	7.33±2.67	6.83±2.55	5.83±2.35	4.67±2.64	4.25±2.53	4.17±3.13	1.83±2.55
p value	0.68	0.08	0.24	0.22	0.14	0.26	0.78

## DISCUSSION

This randomized clinical trial aimed at assessing the effect of applying lyophilized AM to palatal donor site on its pain and healing. In the present study, the pattern of healing and the time of wound repair were evaluated through color match and similarity of the texture of the donor site to the adjacent tissue. In any intervals, the test group showed better results than the control group, which indicated that AM improved and expedited healing process of palatal wound. These two indices studied were also used in similar studies as indicators of healing in palatal donor site [1, 5, 8]. We also used a VAS for assessing color match and tissue texture similar to the study conducted by Keceli, *et al* [8]; by this approach, we tried to evaluate the healing process more objectively and accurately.

AM has been studied as a biological wound dressing material over oral mucosal defects in various intraoral surgeries. Different forms of AM such as cryopreserved [26, 27], hyper-dry [20], fresh [28], and lyophilized (freeze-dried) have been applied in these studies. Based on the results, AM promotes healing and wound epithelialization; it reduces granulation tissue formation in large open wounds with no specific side-effects. 

Velez, *et al* [27], evaluated the efficacy of cryopreserved AM on wound healing after dental implant surgery. They showed that the cryopreserved AM effectively enhances cicatrization and wound healing. The cryopreserved AM supports epithelial growth and subsequently facilitates their migration and reinforces their adhesion. It also decreases pain. 

The efficacy of AM has also been investigated as a biological wound dressing material for mucosal surgical defects in the oropharyngeal region. Researchers assessed the pain score, granulation tissue formation and surface epithelialization of the graft site. The results indicated that AM can be used as an acceptable biological dressing to cover mucosal defects in the oropharyngeal region [28]. The results of the aforementioned studies were in line with ours and showed that the AM could facilitate the palatal donor site healing and the lessen the patients’ pain.

In our study, inflammation was also assessed as an objective indicator of clinical healing. The inflammation on the sidelines of the donor site was more pronounced in the control group at all follow-up visits, although the difference was just significant on day 14 (p<0.05).

GCF is an inflammatory exudate and its assessment has been introduced as a non-invasive tool to monitor periodontal inflammatory responses in different clinical circumstances [29, 30]. Kumar, *et al* [31], studied the anti-inflammatory, anti-infective and clinical properties of AM when used for guided tissue regeneration in contained interdental defects. The interleukin-1β and human b-defensin-2 levels were measured in the gingival crevicular fluid of the test (AM with bone graft) and control (bone graft only) sites. The AM resulted in a significant reduction in the level of interleukin-1β and an insignificant increase in the level of human b-defensin-2 in GCF. The AM demonstrated a marked anti-inflammatory effect, confirming by the results of our study.

In the study of Velez, *et al* [27], the clinical signs or symptoms of inflammation) swelling or redness), pain and abscess formation were also evaluated after placement of AM over surgical wound of implant surgeries. None of the patients showed inflammation or reported adverse events at any time points. No infection was clinically suspected; therefore, no cultures were obtained. Therefore, the authors concluded that AM can reduce inflammation and infection rate, which was similar to what we found that AM application could decrease the risk of infection at the palatal donor site.

In the present study, the overall analgesics intake and the VAS pain scores decreased during the first week after the surgery. However, between-group analysis did not show any significant difference at any intervals. So, the use of AM may have a limited effect on pain. On the other hand, Velez, *et al* [27], reported that significantly fewer patients experienced pain in the AM group, and when present, the severity of pain was significantly less than that in the control group. This difference could be due to the location of the dressing, which was less susceptible to trauma (implant surgical site *vs*. the palatal donor site) and the type of surgeries. 

Arai, *et al* [20], used a hyper-dry AM for treatment of 10 patients with secondary defects in the tongue and buccal mucosa after surgical removal of cancerous or precancerous lesions. The pain scores were recorded in the first week and expressed by patients and categorized as good (none to mild), fair (mild to moderate), and poor (severe). Pain relief was good in five patients and fair in five others. Their results were not consistent with ours, which can be attributed to the location of the dressing (tongue and buccal mucosa vs. the palatal donor site), greater surgical ulcers, and lack of a control group for comparison.

In the study by Khademi, *et al* [28], the pain score was categorized as good (none to mild), fair (mild to moderate), and poor (severe) according to a questionnaire filled out by patients during the first 7 days, post-operatively. The results were favorable in all patients. 

In the present study, clinical photographs and questionnaires were used to assess the efficacy of AM. Clinical photographs have been taken under standardized conditions with similar exposure settings to accurately assess color match, tissue texture and inflammation, and it was technically difficult to obtain standardized shots from the same patient at different visits. Other limitations of our study included absence of histological examination, and variability and problems with different scoring systems. Finally, although care was taken to harvest the graft with a uniform thickness, standardization of wound depth and graft thickness was difficult.

Based on the results of this study, the use of AM as a biological dressing can prevent the complications of free gingival graft (such as pain and bleeding) and may accelerate healing. Further randomized controlled clinical trials involving histological, immunological and microbiological examinations with longer follow up visits are necessary to confirm the efficacy of AM as a biological dressing.

## CONFLICTS OF INTEREST:

**None declared.**

## FINANCIAL SUPPORT:

**This research was supported by Tehran University of Medical Sciences and Health Services, grant # 94-04-52-30217.**
